# Sialyl-lactotetra, a Novel Cell Surface Marker of Undifferentiated Human Pluripotent Stem Cells*[Fn FN2]

**DOI:** 10.1074/jbc.M114.568832

**Published:** 2014-05-19

**Authors:** Angela Barone, Karin Säljö, John Benktander, Maria Blomqvist, Jan-Eric Månsson, Bengt R. Johansson, Johan Mölne, Anders Aspegren, Petter Björquist, Michael E. Breimer, Susann Teneberg

**Affiliations:** From the ‡ Institute of Clinical Sciences, Department of Surgery, S-41 345 Göteborg, Sweden,; the § Institute of Biomedicine, Department of Medical Biochemistry and Cell Biology, S-40530 Göteborg, Sweden,; the ¶ Institute of Biomedicine, Department of Clinical Chemistry and Transfusion Medicine, S-413 45 Göteborg, Sweden,; the ‖ Institute of Biomedicine, Department of Pathology, S-413 45 Göteborg, Sweden, and; the ** Cellectis Stem Cells, Cellartis AB, S-413 46 Göteborg, Sweden

**Keywords:** Carbohydrate Structure, Embryonic Stem Cell, Glycobiology, Glycolipid Structure, Mass Spectrometry (MS), Cell Surface Immune Recognition, Gangliosides, Sialyl-lactotetraosylceramide, Sulfatide

## Abstract

Cell surface glycoconjugates are used as markers for undifferentiated pluripotent stem cells. Here, antibody binding and mass spectrometry characterization of acid glycosphingolipids isolated from a large number (1 × 10^9^ cells) of human embryonic stem cell (hESC) lines allowed identification of several novel acid glycosphingolipids, like the gangliosides sialyl-lactotetraosylceramide and sialyl-globotetraosylceramide, and the sulfated glycosphingolipids sulfatide, sulf-lactosylceramide, and sulf-globopentaosylceramide. A high cell surface expression of sialyl-lactotetra on hESC and human induced pluripotent stem cells (hiPSC) was demonstrated by flow cytometry, immunohistochemistry, and electron microscopy, whereas sulfated glycosphingolipids were only found in intracellular compartments. Immunohistochemistry showed distinct cell surface anti-sialyl-lactotetra staining on all seven hESC lines and three hiPSC lines analyzed, whereas no staining of hESC-derived hepatocyte-like or cardiomyocyte-like cells was obtained. Upon differentiation of hiPSC into hepatocyte-like cells, the sialyl-lactotetra epitope was rapidly down-regulated and not detectable after 14 days. These findings identify sialyl-lactotetra as a promising marker of undifferentiated human pluripotent stem cells.

## Teaser

**Background:** Carbohydrate epitopes are often used as markers for characterization of human pluripotent stem cells (hPSC).

**Results:** Sialyl-lactotetra is highly expressed on the cell surface of hPSC lines, and the expression decreased upon early differentiation.

**Conclusion:** Sialyl-lactotetra is a novel marker of undifferentiated human stem cells.

**Significance:** These findings might impact future applications in regenerative medicine.

## Introduction

Transplantation of human embryonic stem cells (hESC)^5^ and human induced pluripotent stem cells (hiPSC), collectively called human pluripotent stem cells (hPSC), have potential for treating a broad variety of human diseases by cell replacement therapy or by regeneration of damaged tissues (1). However, a major concern that may limit the use of hESC or any other allogeneic stem cell population in therapies is the potential immunologic rejection of the cells, or their derivatives, after transplantation into immunocompetent recipients. Thus, before such therapy can become clinically implemented, the molecular characteristics of the cells need to be defined.

The definition and characterization of hPSC is based on a number of cell surface markers, several of which are carbohydrate epitopes (2). Widely used carbohydrate biomarkers for evaluation of pluripotency are the stage-specific embryonic antigen 3 (SSEA-3/globopentaosylceramide; see supplemental Table S1 for glycosphingolipid structures), SSEA-4 (sialyl-globopentaosylceramide), and the tumor rejection antigens TRA-1–60 and TRA-1–81 (2–5). The globo carbohydrate core is only found in glycosphingolipids, and thus SSEA-3 and SSEA-4 belong to this type of glycoconjugates.

Glycosphingolipids are complex amphipathic compounds consisting of a hydrophilic oligosaccharide linked to a hydrophobic ceramide (6). The structures of both components (oligosaccharide and ceramide) vary, resulting in great molecular heterogeneity. To date, over 300 glycosphingolipids with different carbohydrate head groups have been characterized. All mammalian cell membranes contain glycosphingolipids, and they are also present in intracellular compartments, such as the Golgi apparatus, nuclear membrane, and mitochondria. The glycosphingolipids are divided into acid and non-acid glycosphingolipids, where the acid glycosphingolipids are further subdivided into sialic acid containing glycosphingolipids (gangliosides) and sulfate ester conjugated glycosphingolipids (sulfatides). In addition, the glycosphingolipids are classified on the basis of their carbohydrate core structures. In humans the globo (Galα4Gal), lacto (Galβ3GlcNAc), and neolacto (Galβ4GlcNAc) core chains are the most common ones among non-acid glycosphingolipids, whereas the gangliosides have mainly ganglio (Galβ3GalNAc) or neolacto core chains.^6^

There are only a few studies with chemical structural characterization of the glycosphingolipids of hPSC. Glycosphingolipids from crude lipid extracts have been analyzed by antibody binding, flow cytometry, and mass spectrometry (7, 8). Thereby, some globo series glycosphingolipids (globotetraosylceramide, globopentaosylceramide/SSEA-3, and the Globo H hexaosylceramide), type 1 core chain glycosphingolipids (lactotetraosylceramide and fucosyl-lactotetraosylceramide), and gangliosides (GM3, GM1, GD1a, or GD1b, sialyl-globopentaosylceramide/SSEA-4, and disialyl-globopentaosylceramide) were identified in undifferentiated hESC. In a recent study of the total cellular glycome (*N*- and *O*-linked saccharides, glycosaminoglycans and glycosphingolipids) of hESC and hiPSC, a large number of glycosphingolipid-derived glycans were identified (9). However, in this case the interpretation of data was complicated, because the preparations also contained glycosphingolipids derived from the mouse feeder cells. After eliminating glycan structures that were probably of mouse origin, it was concluded that, compared with human somatic cells, the human stem cells had a higher expression of the glycosphingolipids globopentaosylceramide/SSEA-3, sialyl-globopentaosylceramide/SSEA-4, fucosyl-lactotetraosylceramide/SSEA-5, difucosyl-neolactotetraosylceramide, neolactopentaosylcerami de, neolactotetraosylceramide, globotetraosylceramide, the NeuGc-GM1 ganglioside, and gangliotriosylceramide/asialo-GM2.

These previous studies were done on minute amounts of hESC lines (10^5^–10^6^ cells). We have recently isolated total non-acid glycosphingolipid fractions from two hESC lines (SA121 and SA181), grown in a serum- and cell-free system, using relatively large amounts of cells as starting material (1 × 10^9^ cells/cell line). The characterization of the non-acid glycosphingolipids of these two hESC cell lines by antibody binding, mass spectrometry, and proton NMR was recently reported (10). The high number of cells used allowed a higher resolution of the glycosphingolipids present in the hESC lines, because it was also possible to obtain partly purified glycosphingolipid subfractions. Thus, several non-acid glycosphingolipids not previously described in hPSC were identified, such as galactosylceramide, glucosylceramide, lactosylceramide, galabiaosylceramide, globotriaosylceramide, lactotriaosylceramide, type 2 core chain glycosphingolipids (the H type 2 pentaosylceramide, the Le^x^ pentaosylceramide, and Le^y^ hexaosylceramide), and a blood group A type 1 hexaosylceramide.

In this study we have structurally characterized the acid glycosphingolipid fractions isolated from the two hESC lines SA121 and SA181 by antibody binding and mass spectrometry. In addition to the gangliosides previously described in hESC, we identified some novel hESC gangliosides (sialyl-lactotetraosylceramide and sialyl-globotetraosylceramide), and a number of sulfated glycosphingolipids (sulfatide, sulf-lactosylceramide and sulf-globopentaosylceramide). A high expression of the sialyl-lactotetra carbohydrate sequence on the cell surface of hESC and hiPSC was demonstrated by flow cytometry, immunohistochemistry, and electron microscopy. The sialyl-lactotetra expression was lost upon differentiation of hiPSC into hepatocyte-like cells, suggesting that this carbohydrate sequence is a novel marker for undifferentiated human stem cells.

## EXPERIMENTAL PROCEDURES

### 

#### 

##### hESC and hiPSC Lines, Culture Conditions, and Differentiation of hiPSC

The hESC lines were derived and characterized as previously described (11, 12). The hiPSC lines ChiPSC-7, ChiPSC-9, and P11012 were Cellectis in house human induced pluripotent stem cells derived by the retroviral transduction technique, whereas the derivation of ChiPSC-4 was done as described (13).

The hiPSC lines (ChiPSC-4, ChiPSC-7, ChiPSC-9, and P11012) and four of the hESC lines (SA002, SA121, SA181, and AS038) were cultured under feeder-free conditions (13), whereas the remaining three hESC lines (SA001, SA348m, and SA461) were cultured on mitomycin-C-inactivated mouse embryoid fibroblast feeder layers.

Differentiation of the hiPSC line ChiPSC-4 into hepatocyte-like cells was done as described (13). hESC differentiation into cardiomyocyte-like cells was performed as described (12).

##### Isolation of Acid Glycosphingolipids from hESC

For isolation of glycosphingolipids, the two hECS lines SA121 and SA181 were cultured under feeder-free conditions and harvested, as previously described (10, 14). The cell pellets obtained were stored at −80 °C.

Acid glycosphingolipids were isolated from the two hESC lines as described by Barone *et al.* (10). In each case, ∼2.5 mg of total acid glycosphingolipid fractions was obtained from 1 × 10^9^ cells. These fractions were structurally characterized by thin layer chromatography, binding of monoclonal antibodies, and mass spectrometry. Thereafter, partly purified subfractions were obtained by separation of the acid glycosphingolipids on Iatrobeads (Iatron Laboratories, Tokyo, Japan) columns (0.5 g) and eluted with increasing amounts of methanol in chloroform. Three subfractions (designated fractions 121A, 121B, and 121C and fractions 181A, 181B, and 181C, respectively) were in each case obtained after pooling. These subfractions were further characterized by antibody binding and mass spectrometry.

##### Chromatogram Binding Assays

The reference glycosphingolipids were isolated and characterized by mass spectrometry and proton NMR as described (15). Thin layer chromatography was done on aluminum- or glass-backed silica gel 60 high performance thin layer chromatography plates (Merck). Glycosphingolipid mixtures (40 μg)or pure compounds (2–8 μg) were eluted using chloroform/methanol/water (60:35:8, v/v/v) as a solvent system. Glycosphingolipids were detected by the anisaldehyde reagent (15) or the resorcinol reagent (16).

The mouse monoclonal antibodies tested for binding to the acid glycosphingolipids of hESC in the chromatogram binding assay are given in supplemental Table S2. Binding of antibodies to glycosphingolipids separated on thin layer chromatograms was performed as described by Barone *et al.* (10). In short, glycosphingolipids were separated on aluminum-backed thin layer plates, and after drying the chromatograms were dipped for 1 min in diethylether/*n*-hexane (1:5, v/v) containing 0.5% (w/v) polyisobutylmethacrylate (Sigma-Aldrich) for 1 min. Thereafter, the chromatograms were soaked in PBS, pH 7.3, containing 2% bovine serum albumin and 0.1% NaN_3_ (solution A) for 2 h at room temperature. Suspensions of monoclonal antibodies (the dilutions used for each antibody are given in supplemental Table S2) were gently sprinkled over the chromatograms, followed by incubation for 2 h at room temperature. After washing with PBS followed a second 2-h incubation with ^125^I-labeled rabbit anti-mouse antibodies (DakoCytomation Norden A/S, Glostrup, Denmark) (labeled by the Iodogen method according to the manufacturer's (Pierce) instructions), diluted to 2 × 10^6^ cpm/ml in solution A. Finally, the plates were washed six times with PBS. Dried chromatograms were autoradiographed for 12–24 h using XAR-5 x-ray films (Eastman Kodak).

##### LC-ESI/MS and ESI/MS/MS

The glycosphingolipids (dissolved in methanol/acetonitrile 75:25, v/v) were separated on a 200 × 0.150-mm column, packed in-house with 5-μm polyamine II particles (YMC Europe GMBH, Dinslaken, Germany), and eluted with a water gradient (A: 100% acetonitrile; B: 10 mm ammonium bicarbonate). Samples were analyzed on an LTQ linear quadrupole ion trap mass spectrometer (Thermo Electron) by LC-ESI/MS at −3.5 kV. Full-scan (*m*/*z* 500–1800, two microscans, maximum of 100 ms, target value of 30 000) was performed, followed by data-dependent MS^2^ scans (two microscans, maximum of 100 ms, target value of 10 000) with normalized collision energy of 35%, an isolation window of 2.5 units, an activation *q* = 0.25, and an activation time of 30 ms.

##### Flow Cytometry

Expression of cell surface antigens was evaluated by flow cytometry. The hiPSC lines (ChiPSC-4, ChiPSC-7, ChiPSC-9, and P11012) and hESC lines (SA121, SA181, and AS038) analyzed were cultured under feeder-free conditions. Single cell suspensions (∼2 × 10^5^ cells/tube) were prepared using TrypLE Select (Invitrogen) and washed with PBS containing 2% FCS (FCS/PBS). Thereafter, the cell suspensions were incubated with primary antibodies, or their isotype controls, diluted in FCS/PBS, for 30 min at 4 °C. Duplicate samples were prepared, and the expression was normalized against an internal negative control consisting of secondary antibody of corresponding isotype and isotype controls to account for day to day variations and balance discrepancies between sample preparations. After washings followed incubation with FITC-conjugated secondary antibodies of corresponding isotype, diluted in FCS/PBS, for 30 min at 4 °C. The stained cells were suspended in 200 μl of FCS/PBS or 0.5% paraformaldehyde and analyzed by a FACSCalibur^TM^ flow cytometer (Becton Dickinson). Fluorescence signals from 20,000 cells were recorded and analyzed by the CellQuest pro (Becton Dickinson) and FlowJo software. The cell population was gated to exclude debris and dead cells on the basis of their forward and side scatter characteristics.

The primary antibodies used were anti-SSEA-4 (MC-813-70 clone; 1:50; eBioscience), hES cellect^TM^ (HES 5:3 clone; 1:5; Cellartis AB, Göteborg, Sweden), anti-TRA-1–60 (TRA-1–60 clone; 1:100; eBioscience), anti-SSEA-3 (MC-631 clone; 1:200; eBioscience), anti-sialyl-lactotetra (TR4 clone; 1:100 (17)), anti-sialyl-neolactotetra (LM1:1a clone; 1:100 (18)), and anti-SO_3_-Galβ (Sulf-1; 1:100 (19)). The secondary antibodies used were FITC anti-mouse-IgG (1:100; eBioscience), FITC anti-mouse-IgM (1:60; Santa Cruz), and FITC anti-rat-IgM (1:200; eBioscience). Isotype control for FITC mouse-IgG was ab37356 (1:50; Abcam) and for FITC mouse-IgM ab91546 (1:8; Abcam). The secondary antibody only was used as negative control for rat IgM.

##### Immunohistochemistry

Immunohistochemical analyses were performed as described (12). The hiPSC lines (ChiPSC-4, ChiPSC-7, and ChiPSC-9), and four of the hESC lines (SA002, SA121, SA181, and AS038) were cultured under feeder-free conditions, whereas the remaining three hESC lines (SA001, SA348, and SA461) were cultured on mitomycin-C-inactivated mouse embryoid fibroblast feeder layers. The primary antibodies used were anti-sialyl-lactotetra (TR4 clone; 1:500 dilution (17)), anti-sialyl-neolactotetra (LM1:1a clone; 1:500 dilution (18)), anti-SO_3_-Gal (Sulf-1; 1:200 dilution (19)), and anti-human TRA-1–60 (TRA-1–60 clone; 1:100 dilution; eBioscience). Dako EnVision detection kit peroxidase/DAB (Dako) was used for detection of bound antibodies.

##### Electron Microscopy: Sample Preparations and Examination

Human embryonic stem cells (SA121 and SA181) layers were fixed in the culture vessel with 2% paraformaldehyde and 0.2% glutaraldehyde in 0.1 m phosphate buffer for 2 h. After rinsing twice with PBS and treatment with PBS/glycine (0.02 m glycine, 0.02% sodium azide) for 10 min, layers were harvested by scraping with a rubber policeman. Alternatively, cells were first released by protease treatment (TrypLE^TM^ DPBS, 1 mm EDTA; Invitrogen) and thereafter fixed in the same fixative as above. The harvested materials were enriched by centrifugation in fluid gelatin at a final concentration of 12% that was allowed to solidify at 4 °C. Further preparation followed the Tokuyasu protocol (20), with appropriate modifications. Small cubes were cut with a razor blade and subjected to 2.3 m sucrose infusion in a rotary stand for >72 h. Cubes were frozen by immersion in LN_2_ and cryosectioned at −110 °C and section setting at 50 nm in a Leica UC6 cryo-ultramicrotome (Leica Microsystems, Vienna, Austria) fitted with a 35° diamond cryo-knife (Diatome, Biel, Switzerland). Sections were collected on copper grids with carbon coated Formvar support film.

For comparison purposes, frozen samples of scraped cell layers were also thawed, rinsed in buffer to remove sucrose, and refixed chemically with 2.5% glutaraldehyde. They were post-fixed with 1% OsO_4_ with 1% potassium hexacyanoferrate, contrasted *en bloc* with 0.5% uranyl acetate, and further processed according to routines into epoxy resin embedding. Electron microscopy sections were counterstained with uranyl acetate and lead citrate.

The electron microscopy sections were examined in a LEO 912AB Omega (Carl Zeiss) transmission electron microscope with a LaB_6_ electron source. Digital imaging was achieved with a Veleta CCD camera (2K × 2K Olympus Soft Imaging Solutions, Münster, Germany); the camera and microscope were run under the iTEM software (Olympus Soft Imaging Solutions). The original image files in TIF format were further processed with Adobe Photoshop and Illustrator.

##### Immunogold Procedures

Thawed cryosections were floated on drops of melted 2% gelatin followed by 0.1% glycine in PBS. After rinsing in PBS, 1% BSA, the cryosections were blocked with PBS, 1% BSA complemented with 2% goat serum to block nonspecific binding sites and then exposed to 5-μl droplets with primary antibodies diluted in PBS, 1% BSA for 20 min, followed by five rinses with PBS. The primary antibodies used were anti-sialyl-lactotetra (TR4 clone; 1:200 (17)), anti-sialyl-neolactotetra (LM1:1a clone; 1:200 (18)), and anti-sulfatide (O4 clone; 1:50; R&D Systems). The second 20-min incubation step was with goat anti-mouse IgM/18 nm gold (1:10; Abcam) to localize mouse monoclonals. After rinsing, sections were stabilized with 1% glutaraldehyde in PBS for 5 min. Sections were contrasted with 2% uranyl oxaloacetate, pH 7.0, followed by infiltration with ice-cold 1.8% methyl cellulose and 0.4% uranylacetate and air drying.

##### hESC Protein Binding Assay

Human embryonic stem cells from the two cell lines (SA121 and SA181) were suspended in standard lysis buffer (50 mm Tris-HCl, pH 8,0, 150 mm NaCl, and 1% (v/v) Triton X-100) supplemented with a EDTA-free protease inhibitor mixture (Roche) and incubated on ice for 30 min, followed by centrifugation. The supernatants were collected and mixed with equal amounts of 2 × LDS sample buffer (NuPAGE) supplemented with 100 mm dithiothreitol for reducing conditions and heated at 95 °C for 5 min.

Cell lysates (13 μg from each cell line) were separated by electrophoresis on 4–12% Bis-Tris gels (NuPAGE) with precision protein standards (Bio-Rad) as markers and either stained by ImperialTM Protein Stain (Thermo Scientific) or transferred to nitrocellulose (0,2 μm) membranes (Bio-Rad). The blots was blocked with PBS containing 0.1% Tween 20 and 2% BSA (Solution B) for 1 h and thereafter incubated with anti-TRA-1–60 antibodies (TRA-1–60 clone; 1:200; Abcam) or anti-sialyl-lactotetra antibodies ((TR4 clone; 1:500 (17)) diluted in solution B overnight at 4 °C. After washing with solution B followed a second incubation with alkaline phosphate-conjugated goat anti-mouse antibodies (1:3000; Southern Biotech) diluted in solution B for 1 h at room temperature. Finally the membranes were washed with solution B and developed by using nitroblue tetrazolium 5-bromo-4-chloro-3-indolyl phosphate (Southern Biotech).

## RESULTS

### 

#### 

##### Structural Characterization Identifies Sulfatide, Sulf-lactosylceramide, Sulf-globopentaosylceramide, Sialyl-globotetraosylceramide, and Sialyl-lactotetraosylceramide as Novel Glycosphingolipids of hESC Lines

**FIGURE 1.**
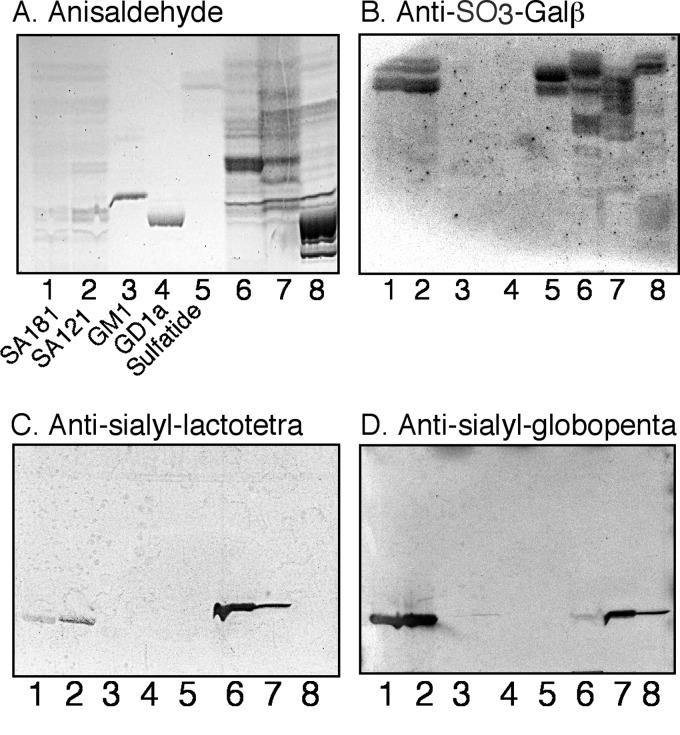
**Characterization of the acid glycosphingolipids of hESC by binding of monoclonal antibodies.** *A–D*, thin layer chromatogram after detection with anisaldehyde (*A*), and autoradiograms obtained by binding of the monoclonal antibodies directed against SO_3_-3Galβ (*B*), sialyl-lactotetra (*C*), and sialyl-globopenta/SSEA-4 (*D*). *Lane 1*, total acid glycosphingolipids of hESC line SA121, 40 μg: *lane 2*, total acid glycosphingolipids of hESC line SA181, 40 μg: *lane 3*, reference GM1 ganglioside (Galβ3GalNAcβ4(NeuAcα3)Galβ4Glcβ1Cer), 4 μg; *lane 4*, reference GD1a ganglioside (NeuAcα3 Galβ3GalNAcβ4(NeuAcα3)Galβ4Glcβ1Cer), 4 μg; *lane 5*, reference sulfatide (SO_3_-3Galβ1Cer), 2 μg; *lane 6*, total acid glycosphingolipids of human hepatoma, 40 μg; *lane 7*, total acid glycosphingolipids of human lung cancer liver metastasis, 40 μg; *lane 8*, total acid glycosphingolipids of calf brain, 40 μg.

Approximately 2.5 mg of total acid glycosphingolipids were obtained from each of the hESC lines SA121 and SA181. When analyzed by thin layer chromatography and chemical detection, both acid fractions (Fig. 1*A*, *lanes 1* and *2*) had a number of faint fast migrating bands, and a number of bands migrating below the level of the reference GM1 ganglioside (Fig. 1*A*, *lane 3*).

At first, the binding of a number of monoclonal antibodies to the acid fractions isolated from hESC lines SA121 and SA181 was tested. The antibodies used are listed in supplemental Table S2. In total the binding of 13 monoclonal antibodies directed against acid carbohydrate epitopes was evaluated. However, only three of these antibodies bound to the hESC acid glycosphingolipids (Fig. 1).

The Sulf-1 antibody binds to glycoconjugates with terminal SO_3_-3Galβ. This antibody bound to two sets of double bands migrating just above and below the level of reference sulfatide (Fig. 1*B*, *lane 5*) in the hESC acid glycosphingolipid fractions (Fig. 1*B*, *lanes 1* and *2*), indicating the presence of SO_3_-3Galβ-terminated glycosphingolipids, as sulfatide and sulfated lactosylceramide.

There was also a distinct binding of the monoclonal antibodies directed against the sialyl-lactotetra and sialyl-globopenta/SSEA4 epitopes in the slow migrating region of the acid fractions (Fig. 1, *C* and *D*), suggesting the presence of sialyl-lactotetraosylceramide and sialyl-globopentaosylceramide. There was also a distinct binding of the anti-sialyl-lactotetra antibody to the two reference acid glycosphingolipid fractions of human cancers (Fig. 1*C*, *lanes 6* and *7*).

In addition, there were a number of cases when antibodies tested showed appropriate reactivity with the reference glycosphingolipids used but did not bind to the acid glycosphingolipids of hESC. Thus, no hESC binding was obtained with monoclonal antibodies directed against the sialyl-Le^a^ epitope; the sialyl-Le^x^ epitope; the HNK-1 epitope; the gangliosides GD1a, GM1, GM2, and GD3; and gangliosides with neolacto core and terminal NeuAcα3 or NeuAcα6 (supplemental Table S2).

**FIGURE 2.**
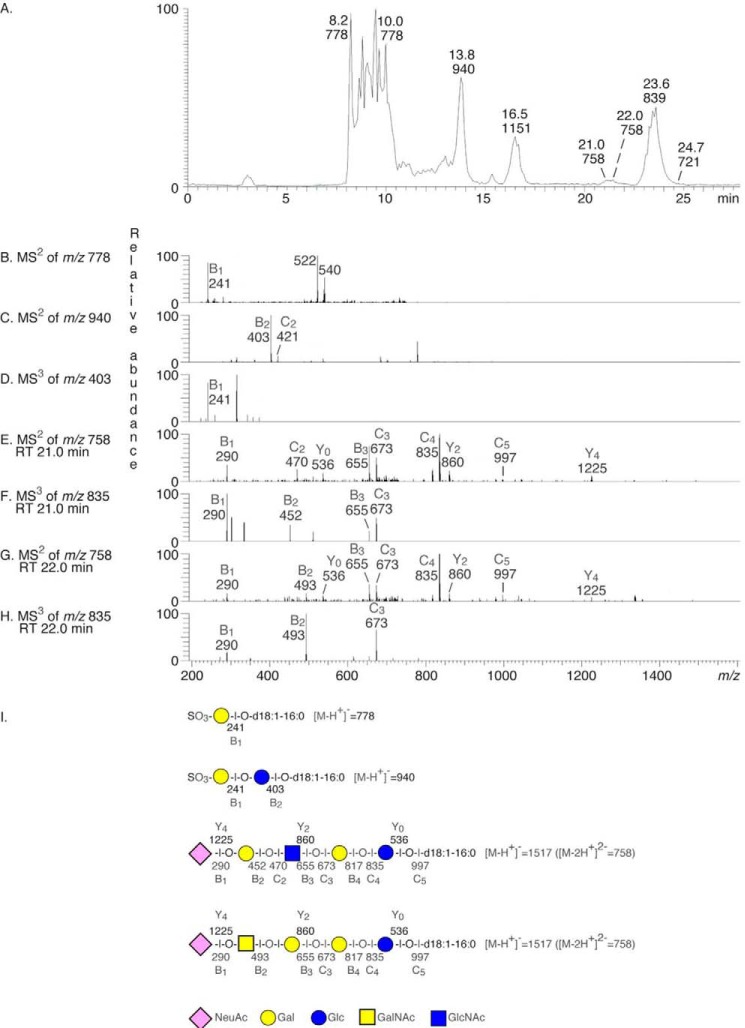
**Characterization of the acid glycosphingolipids of hESC by mass spectrometry.** *A*, base peak chromatogram from LC-ESI/MS of the total acid glycosphingolipids from hESC line SA181. *B*, MS^2^ of the molecular ion at *m*/*z* 778 (retention time, 8.2 min) gave a B_1_ ion at *m*/*z* 241, confirming a terminal SO_3_-Hex. The ions at *m*/*z* 540 and 522 are due to loss of the fatty acyl from the molecular ion (38). *C*, MS^2^ of the molecular ion at *m*/*z* 940 (retention time, 13.7 min) gave a B_2_ ion at *m*/*z* 403, and a C_2_ ion at *m*/*z* 421, demonstrating a terminal SO_3_-Hex-Hex sequence. *D*, MS^3^ of the fragment ion at *m*/*z* 403 gave a B_1_ ion at *m*/*z* 241, confirming a terminal SO_3_-Hex. *E*, MS^2^ of the molecular ion at *m*/*z* 758 (retention time, 21.0 min) gave a series of B and C ions (B_1_ at *m*/*z* 290, C_2_ at *m*/*z* 470, B_3_ at *m*/*z* 655, C_3_ at *m*/*z* 673, B_4_ at *m*/*z* 817, C_4_ at *m*/*z* 835, and B_5_ at *m*/*z* 979) and a series of Y ions (Y_0_ at *m*/*z* 536, Y_2_ at *m*/*z* 860, and Y_4_ at *m*/*z* 1225), demonstrating a glycosphingolipid with NeuAc-Hex-HexNAc-Hex-Hex carbohydrate sequence and d18:1–16:0 ceramide. *F*, MS^3^ of the fragment ion at *m*/*z* 835 (retention time, 21.0) min gave a B_2_ ion at *m*/*z* 452, confirming a terminal NeuAc-Hex sequence. *G*, MS^2^ of the molecular ion at *m*/*z* 758 (retention time, 22.0 min) gave a B_1_ ion at *m*/*z* 290, indicating a terminal NeuAc, which was accompanied by a B_2_ ion at *m*/*z* 493, demonstrating a terminal NeuAc-HexNAc sequence. There was also a series of B and C type ions (B_3_ at *m*/*z* 655, C_3_ at *m*/*z* 673, B_4_ at *m*/*z* 817, C_4_ at *m*/*z* 835, and C_5_ at *m*/*z* 997) and Y type ions at *m*/*z* 536 (Y_o_) and *m*/*z* 860 (Y_2_). Taken together, this demonstrated a ganglioside with NeuAc-HexNAc-Hex-Hex-Hex sequence with d18:1–16:0 ceramide, *i.e.* sialyl-globotetraosylceramide. *H*, MS^3^ of the fragment ion at *m*/*z* 835 (retention time, 22.0 min) gave a B_2_ ion at *m*/*z* 493, confirming a terminal NeuAc-HexNAc sequence. *I*, interpretation formulae showing the deduced glycosphingolipid sequences.

Next, the total acid glycosphingolipid fractions were analyzed by LC-ESI/MS. The base peak chromatograms obtained from the total acid glycosphingolipid fractions of the two hESC lines (exemplified in Fig. 2*A*) had a number of both singly charged ([M-H^+^]^−^) and doubly charged ([M-2H^+^]^2−^ ions) molecular ions, giving information on the molecular masses of the glycosphingolipids. The dominating ions were found at *m*/*z* 778 and *m*/*z* 940. In addition, molecular ions at *m*/*z* 1151, 721, 758, and 839 were present. The identity of the glycosphingolipids was obtained by MS/MS (MS^2^), from which the carbohydrate sequence and ceramide composition were deduced.

**FIGURE 3.**
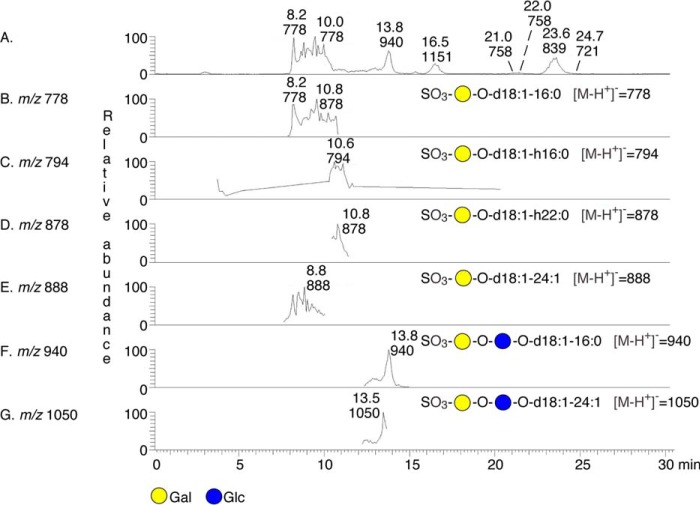
**Characterization of the acid glycosphingolipids of hESC by mass spectrometry.** *A*, base peak chromatogram from LC-ESI/MS of the total acid glycosphingolipids from hESC line SA181. The interpretation formulae show the deduced glycosphingolipid sequences. *B*, mass chromatogram of *m*/*z* 778. *C*, mass chromatogram of *m*/*z* 794. *D*, mass chromatogram of *m*/*z* 878. *E*, mass chromatogram of *m*/*z* 888. *F*, mass chromatogram of *m*/*z* 940. *G*, mass chromatogram of *m*/*z* 1050.

The molecular ion at *m*/*z* 778 indicated sulfatide with d18:1–16:0 ceramide, and the SO_3_-Hex terminal was confirmed by MS^2^ (Fig. 2*B*). A sulfated glycosphingolipid with two Hex and d18:1–16:0 ceramide, as sulfated lactosylceramide, was suggested by the molecular ion at *m*/*z* 940, and this was confirmed by MS^2^ and MS^3^ (Fig. 2*C*). Upon further examination of mass chromatograms, a number of sulfatides with d18:1-h16:0, d18:1-h22:0, and d18:1–24:1 ceramides^7^ were also found, along with sulfated lactosylceramide with d18:1–24:1 ceramide (Fig. 3).

**FIGURE 4.**
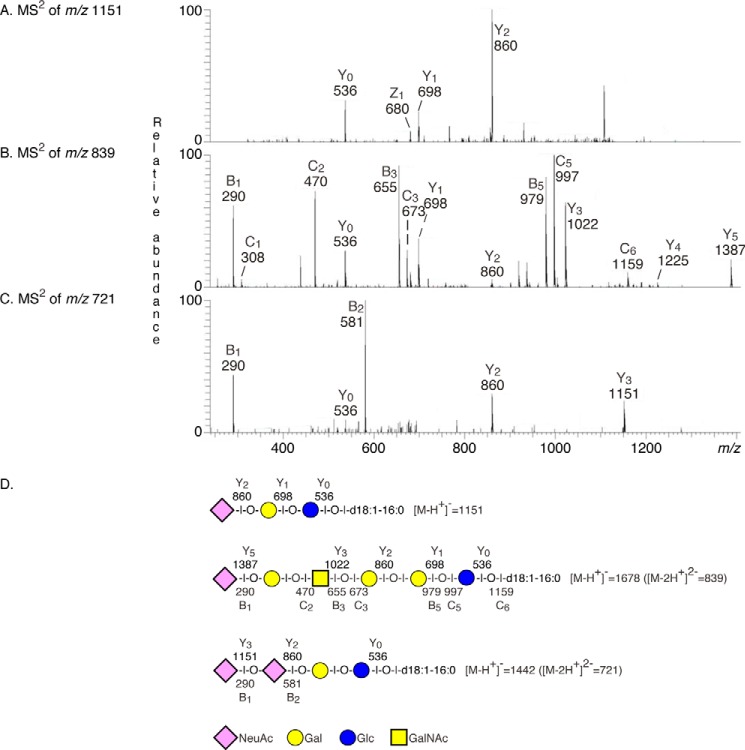
**Characterization of the acid glycosphingolipids of hESC by mass spectrometry.** LC-ESI/MS of the total acid glycosphingolipids from hESC line SA181. *A*, MS^2^ of the molecular ion at *m*/*z* 1151 (retention time, 16.5 min) gave a series of Y ions (Y_0_ at *m*/*z* 536, Y_1_ at *m*/*z* 698, and Y_2_ at *m*/*z* 860), demonstrating a glycosphingolipid with NeuAc-Hex-Hex carbohydrate sequence and d18:1–16:0 ceramide, *i.e.* the GM3 ganglioside. *B*, MS^2^ of the molecular ion at *m*/*z* 839 (retention time, 23.7 min) gave a series of B and C ions (B_1_ at *m*/*z* 290, C_2_ at *m*/*z* 470, B_3_ at *m*/*z* 655, C_3_ at *m*/*z* 673, B_5_ at *m*/*z* 979, C_5_ at *m*/*z* 997, and C_6_ at *m*/*z* 1159), which, along with the series of Y ions (Y_0_ at *m*/*z* 536, Y_1_ at *m*/*z* 698, Y_2_ at *m*/*z* 860, Y_3_ at *m*/*z* 1022, Y_4_ at *m*/*z* 1225, and Y_5_ at *m*/*z* 1387), allowed identification of NeuAc-Hex-HexNAc-Hex-Hex-Hex glycosphingolipid with d18:1–16:0 ceramide, *i.e.* sialyl-globopentaosylceramide. *C*, MS^2^ of the molecular ion at *m*/*z* 721 (retention time, 24.9 min) gave B ions (B_1_ at *m*/*z* 290 and B_2_ at *m*/*z* 581) and a series of Y ions (Y_0_ at *m*/*z* 536, Y_2_ at *m*/*z* 860, and Y_3_ at *m*/*z* 1151), demonstrating a glycosphingolipid with NeuAc-NeuAc-Hex-Hex carbohydrate sequence and d18:1–16:0 ceramide, *i.e.* the GD3 ganglioside. *D*, interpretation formulae showing the deduced glycosphingolipid sequences.

A number of gangliosides previously described in hESC, *i.e.* the GM3 ganglioside (*m*/*z* 1151), sialyl-globopentaosylceramide (*m*/*z* 839), and the GD3 ganglioside (*m*/*z* 721), were also identified by MS^2^ (Fig. 4). In addition, the base peak chromatograms had two ions at *m*/*z* 758, eluting at separate retention times (21.0 and 22.0 min, respectively), indicating the presence of two different gangliosides, both with one NeuAc, one HexNAc, and three Hex, and d18:1–16:0 ceramide. MS^2^ of the ion eluting at 21.0 min identified a ganglioside with NeuAc-Hex-HexNAc-Hex-Hex sequence (Fig. 2, *E* and *F*). MS^3^ of the fragment ion at *m*/*z* 835 gave a B_2_ ion at *m*/*z* 452 confirming a terminal NeuAc-Hex sequence (Fig. 2*F*). The ganglioside sialyl-lactotetraosylceramide was thus identified by these MS^2^ and MS^3^ spectral features, taken together with the distinct binding of anti-sialyl-lactotetra antibodies to the acid glycosphingolipids of hESC (Fig. 1*C*) and the absence of binding of antibodies directed against sialyl-neolactotetra.

The MS^2^ spectrum of the molecular ion at *m*/*z* 758 at a retention time of 22.0 min (Fig. 2, *G* and *H*) was significantly different and demonstrated a ganglioside with NeuAc-HexNAc-Hex-Hex-Hex sequence, *i.e.* sialyl-globotetraosylceramide. Here the B_2_ ion at *m*/*z* 493, obtained by MS^3^ of the fragment ion at *m*/*z* 835, confirmed the terminal NeuAc-HexNAc sequence (Fig. 2*H*).

**FIGURE 5.**
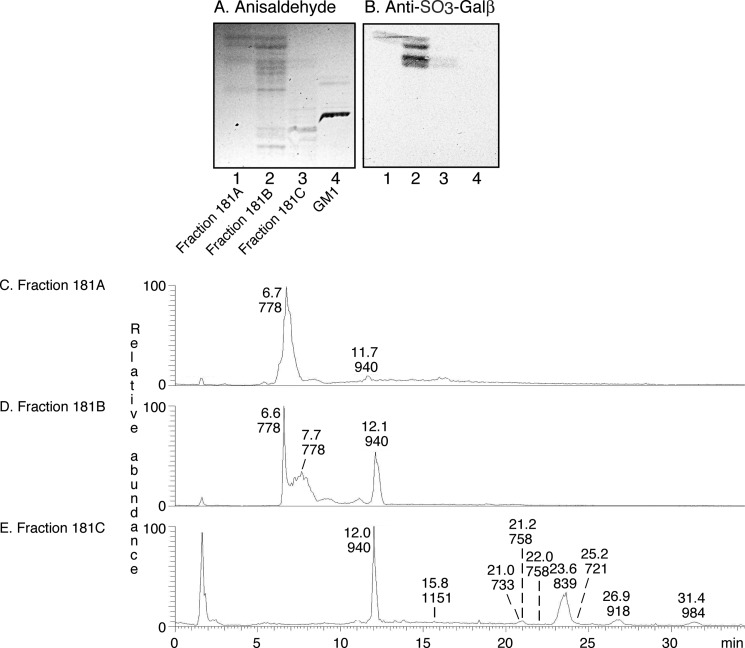
**Characterization of the acid subfractions from hESC by binding of monoclonal antibodies, and by mass spectrometry.** *A* and *B*, thin layer chromatograms after detection with anisaldehyde (*A*) and autoradiogram obtained by binding of the monoclonal anti-SO_3_-3Galβ antibodies (*B*). *Lane 1*, fraction 181A of hESC line SA181, 5 μg; *lane 2*, fraction 181B of hESC line SA181, 5 μg; *lane 3*, fraction 181C of hESC line SA181, 5 μg; *lane 4*, reference GM1 ganglioside (Galβ3GalNAcβ4(NeuAcα3)Galβ4Glcβ1Cer), 8 μg. *C*, base peak chromatogram from LC-ESI/MS of fraction 181A. *D*, base peak chromatogram from LC-ESI/MS of fraction 181B. *E*, base peak chromatogram from LC-ESI/MS of fraction 181C.

Because the molecular ions of sulfated mono- and dihexosylceramide were very dominating in the base peak chromatograms of the total acid glycosphingolipid fractions, the acid glycosphingolipids of the two cell lines were next further separated, to also identify minor compounds. After pooling, three subfractions from each cell line were obtained (exemplified in Fig. 5*A*). The first two fractions (denoted fractions 121A/181A and 121B/181B, respectively) contained fast migrating compounds that were identified as sulfatide and sulf-lactosylceramide, by binding of the Sulf-1 antibody (Fig. 5*B*) and LC-ESI/MS (Fig. 5, *C* and *D*).

**FIGURE 6.**
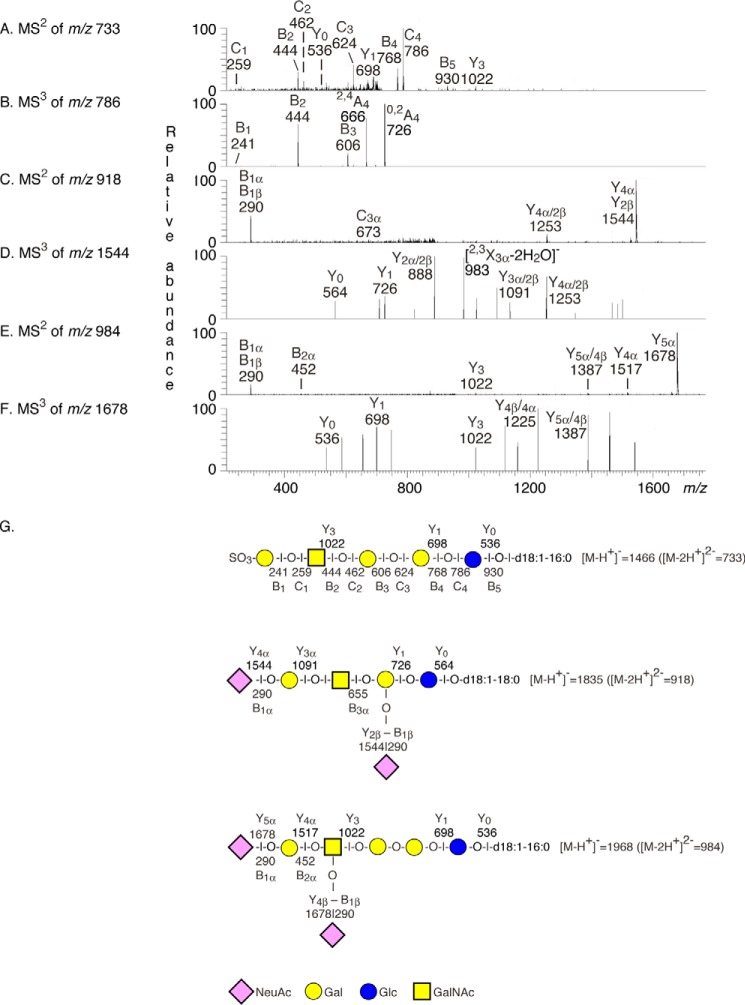
**Characterization of fraction 181C by mass spectrometry, and LC-ESI/MS of fraction 181C from hESC line SA181.** *A*, MS^2^ of the molecular ion at *m*/*z* 733 (retention time, 21.0 min) gave a C_1_ ion at *m*/*z* 259 demonstrating a terminal SO_3_-Hex, and a SO_3_-Hex-HexNAc-Hex-Hex-Hex sequence was demonstrated by the series of B- and C-type ions at *m*/*z* 444 (B_2_), *m*/*z* 462 (C_2_), *m*/*z* 624 (C_3_), *m*/*z* 768 (B_4_), *m*/*z* 786 (C_4_), and *m*/*z* 930 (C_5_). This was corroborated by the series of Y type ions at *m*/*z* 536 (Y_o_), *m*/*z* 698 (Y_1_), and *m*/*z* 1022 (Y_3_), also demonstrating a d18:1–16:0 ceramide. Taken together these features identified a sulf-globopentaosylceramide. *B*, MS^3^ of the fragment ion at *m*/*z* 786 supported the suggested terminal SO_3_-Hex-HexNAc-Hex-Hex sequence. *C*, MS^2^ of the molecular ion at *m*/*z* 918 (retention time, 21.5 min) gave a number of B and Y type ions identifying a ganglioside with NeuAc-Hex-HexNAc(NeuAc)Hex-Hex sequence and d18:1–18:0 ceramide, *i.e.* the GD1a ganglioside (39). *D*, MS^3^ of the fragment ion at *m*/*z* 1544 supported the suggested NeuAc-Hex-HexNAcHex-Hex/Hex-HexNAc(NeuAc)Hex-Hex sequence. *E*, MS^2^ of the molecular ion at *m*/*z* 984 (retention time, 31.5 min) demonstrated a ganglioside with two NeuAc, four Hex, and one HexNAc and d18:1–16:0 ceramide. A terminal NeuAc-Hex sequence was indicated by the B_2α_ ion at *m*/*z* 452 and the Y_4α_ ion at *m*/*z* 1517. *F*, MS^3^ of the fragment ion at *m*/*z* 1678 gave a series of Y ions, which, together with the B_2α_ ion and the Y_4α_ ion in *E*, allowed identification of a ganglioside with NeuAc-Hex-(NeuAc-)HexNAc-Hex-Hex-Hex sequence, as the disialyl-globopentaosylceramide (40). *G*, interpretation formulae showing the deduced glycosphingolipid sequences.

The third fractions (denoted fractions 121C and 181C) contained slow migrating compounds that were stained by resorcinol demonstrating the presence of sialic acids, *i.e.* gangliosides (data not shown). The base peak chromatogram of fraction 181C (Fig. 5*E*) had molecular ions corresponding to sulf-lactosylceramide, sialyl-lactotetraosylceramide, sialyl-globotetraosylceramide, the GD3 ganglioside, and sialyl-globopentaosylceramide, as above. In addition, a number of minor molecular ions were found at *m*/*z* 733, 918, and 984, and by MS^2^ of these ions sulf-globopentaosylceramide, the GD1a ganglioside and disialyl-globopentaosylceramide were identified (Fig. 6).

In summary, by antibody binding and mass spectrometry, a number of novel acid glycosphingolipids of hESC lines (sulfatide, sulf-lactosylceramide, sialyl-lactotetraosylceramide, sialyl-globotetraosylceramide, and sulf-globopentaosylceramide) were characterized, along with previously identified hESC gangliosides. The acid glycosphingolipids of the two hESC lines identified in this study are summarized in Table 1.

**TABLE 1:** **hESC glycosphingolipid structures identified in this study**

Carbohydrate sequence by mass spectrometry	Candidate glycosphingolipid	Trivial name
**Previously identified in hESC**		
NeuAc-Hex-Hex	NeuAcα3Galβ4Glcβ1Cer	GM3
NeuAc-NeuAc-Hex-Hex	NeuAcα8NeuAcα3Galβ4Glcβ1Cer	GD3
NeuAc-Hex-HexNAc-Hex-Hex-Hex	NeuAcα3Galβ3GalNAcβ3Galα4Galβ4Glcβ1Cer	Sialyl-globopenta
NeuAc-Hex-(NeuAc-)HexNAc-Hex-Hex-Hex	NeuAcα3Galβ3 (NeuAcα6)GalNAcβ3Galα4Galβ4Glcβ1Cer	Disialyl-globopenta
NeuAc-Hex-HexNAc-(NeuAc-)Hex-Hex	NeuAcα3Galβ3GalNAcβ4 (NeuAcα3)Galβ4Glcβ1Cer	GD1a

**Novel hESC glycosphingolipids**		
SO_3_-Hex	SO_3_-3Galβ1Cer	Sulfatide
SO_3_-Hex-Hex	SO_3_-3Galβ4Glcβ1Cer	Sulf-LacCer
NeuAc-HexNAc-Hex-Hex-Hex	NeuAcα3GalNAcβ3Galα4Galβ4Glcβ1Cer	Sialyl-globotetra
NeuAc-Hex-HexNAc-Hex-Hex	NeuAcα3Galβ3GlcNAcβ3Galβ4Glcβ1Cer	Sialyl-lactotetra
SO_3_-Hex-HexNAc-Hex-Hex-Hex	SO_3_-3Galβ3GalNAcβ3Galα4Galβ4Glcβ1Cer	Sulf-globopenta

##### The Sialyl-lactotetra Epitope Is Highly Expressed on the Cell Surface of hPSC

**FIGURE 7.**
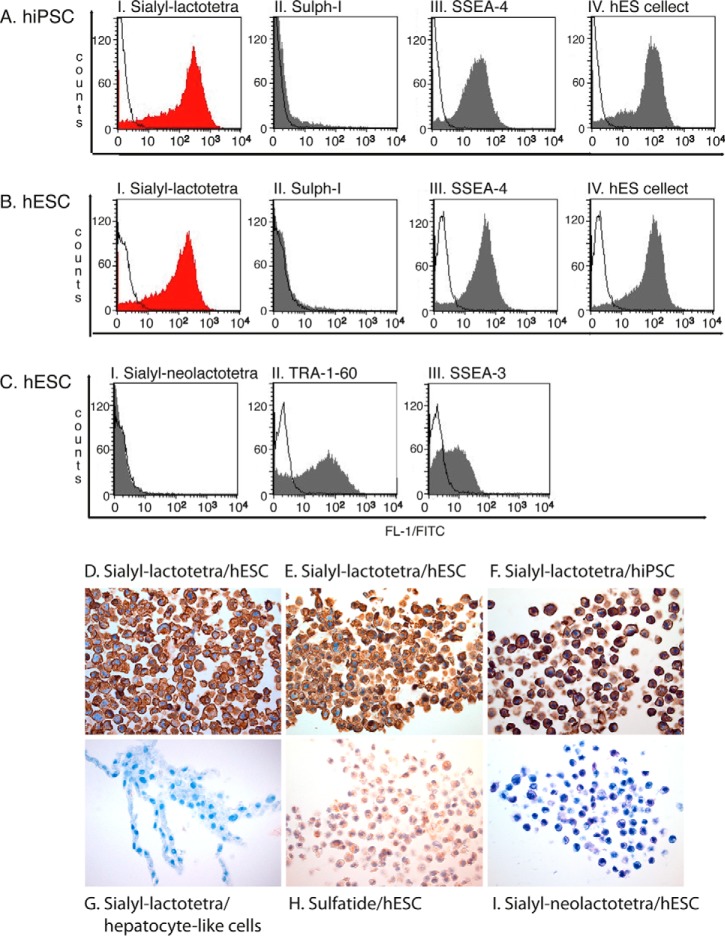
**The sialyl-lactotetra epitope is highly expressed on the cell surface of undifferentiated stem cells.** *A*, representative flow cytometry diagrams of hiPSC incubated with antibodies against sialyl-lactotetra, SO_3_-3Galβ (*Sulph-1*), SSEA-4, and the hES cellect^TM^ marker. *B*, representative flow cytometry diagrams of hESC incubated with antibodies against sialyl-lactotetra, SO_3_-3Galβ (*Sulph-1*), SSEA-4, and the hES cellect^TM^ marker. *C*, representative flow cytometry diagrams of hESC incubated with antibodies against sialyl-neolactotetra, TRA-1–60 and SSEA-3. *Filled red* and *gray* histograms represent positively stained cells. *White histograms* represent control experiments where cells were only stained with secondary antibodies. *D–G*, immunohistochemical investigation with anti-sialyl-lactotetra antibodies of hESC line SA121 (*D*), hESC line SA181 (*E*), hiPSC line ChiPSC-4 (*F*), and hepatocyte-like cells derived from hESC (*G*). All undifferentiated cell lines (*D–F*) show a strong intracellular and surface labeling, whereas the hepatocyte-like cells (*G*) are completely unstained. *H*, immunostaining with anti-SO_3_-3Galβ antibodies of hESC line SA121, showing a weak intracellular staining. *I*, control staining with anti-sialyl-neolactotetra antibodies of hiPSC line ChiPSC-4 is completely negative.

Having identified sulfatide/sulf-lactosylceramide and sialyl-lactotetraosylceramide as novel acid glycosphingolipids of hESC lines, we next examined the expression of these compounds on the cell surface of hESC and on hiPSC by flow cytometry (Fig. 7, *A–C*). As expected, the previously defined stem cell markers SSEA-3, SSEA-4, hES cellect^TM^, and TRA-1–60 were highly expressed on the examined hESC (*n* = 2) and hiPSC (*n* = 4) lines. In addition, almost 95% of the hESC and hiPSC lines expressed high levels of the sialyl-lactotetra epitope on their cell surfaces. In contrast, the structurally related sialyl-neolactotetra epitope was not detectable in either hESC or hiPSC lines. No expression of cell surface sulfatide was detected by flow cytometry.

Next the expression of sialyl-lactotetra was investigated by immunohistochemistry. Here the undifferentiated stem cells (hESC; Fig. 7, *D* and *E*, and hiPSC; Fig. 7*F*) were all strongly positive on the cell surface and in the cytoplasm. In contrast, there was no staining of hepatocyte-like cells (Fig. 7*G*), or cardiomyocyte-like cells (not shown), derived from hESC. In total, the anti-sialyl-lactotetra antibody gave a distinct cell surface staining on all seven hESC lines and three hiPSC lines analyzed. This strongly suggests that sialyl-lactotetra epitope is specifically expressed in undifferentiated human stem cells. The SO_3_-3Galβ binding antibody Sulf-1, on the other hand, gave a weak, diffuse cytoplasmic staining (Fig. 7*H*), whereas no staining of hESC (data not shown) or hiPSC (Fig. 7*I*) lines was obtained with the anti-sialyl-neolactotetra antibody.

**FIGURE 8.**
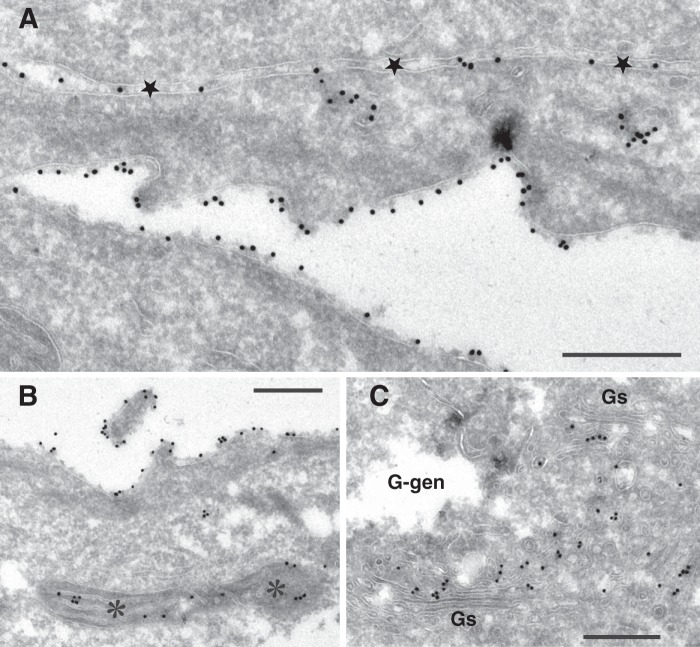
**Subcellular distribution of the sialyl-lactotetra epitope in undifferentiated stem cells.** Electron microscopy of hESC line SA181 immunostained with monoclonal antibodies directed against the sialyl-lactotetra epitope is shown. Also shown is primary antibody binding to cryosections of cells harvested by scraping demonstrated with 18-nm gold-conjugated secondary antibody. *Scale bars*, 0.5 μm. *A*, luminal surface of two cell layers that face each other is richly decorated with immunogold. Note that almost every gold particle is at a distance from the cell membrane (seen as a *white line*), seemingly associated with a patchy surface layer. *Stars* indicate an intercellular cleft between two overlaying cells, also labeled with gold particles. Two intracellular aggregates of gold is seen in the lower of these cells. *B*, selected area demonstrating gold labeling of a mitochondrion (*asterisks*) in addition to surface distribution of label. *C*, central region of cell hitting two Golgi stacks (*Gs*) and vesicle-rich domain in between with obvious gold labeling. The hESC are rich in intracellular glycogen deposits, which on cryosectioning appear as translucent irregular cavities (*G-gen*). The presence of glycogen in these cavities was confirmed by the reference micrograph (Fig. 9*D*).

**FIGURE 9.**
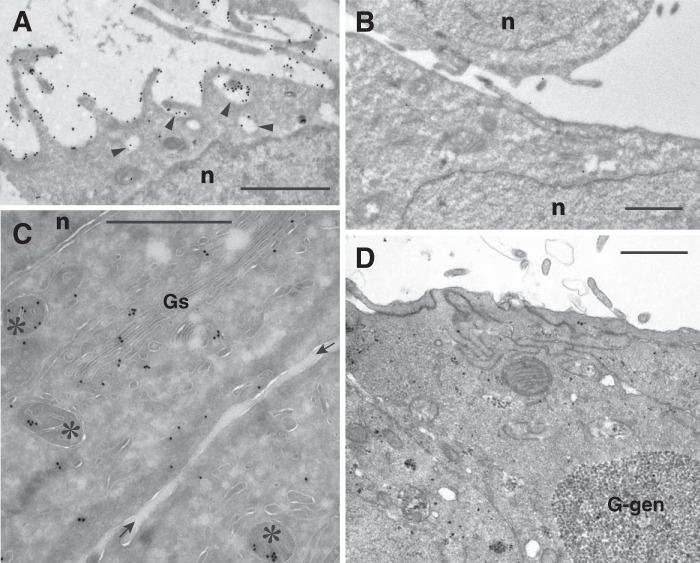
**Electron microscopy of hESC line SA181.** *A–D*, electron microscopy of hESC line SA181 after immunostaining of cryosections (*A–C*) and after plastic re-embedding (*D*). *A*, immunostaining of enzyme-released cells with monoclonal anti-sialyl-lactotetra antibody. The cell surface shows numerous slender projections and invaginations (*arrowheads*) labeled with gold. *n*, nucleus. *B*, control incubation with monoclonal anti-sialyl-neolactotetra antibody followed by secondary 18-nm gold-labeled antibody; scraped cell sheets. A few widely separated gold particles are located at cell surfaces with no intracellular labeling. *n*, nucleus. *C*, immunostaining with the sulfatide binding monoclonal antibody O4 of two opposing cells in sheets after scraping harvest (space between cell sheets indicated with *arrows*). There were no gold particles along cell surface membranes. Labeled mitochondria (*asterisks*) and peripheral parts of Golgi region are shown. *Gs*, Golgi stack. *n*, nucleus. *D*, reference micrograph of cell sheet preparation first subjected to cryoprotection and freezing, then thawing, standard fixation, and plastic embedding. Cells overlap with complex interrelations toward the culture medium (upwards). This preparation mode reveals the coarse granular texture of large (*G-gen*) and small glycogen deposits. *Scale bars*, 1 μm.

The differential subcellular distribution of sialyl-lactotetra and sulfatide demonstrated by immunohistochemistry was further investigated by electron microscopy after immunostaining with the anti-sialyl-lactotetra antibody and the sulfatide-binding antibody O4 (21). The immunocytochemical detection of the sialyl-lactotetra epitope showed a dense labeling of the apical cell surface (toward the culture medium) in hESC cultures fixated and thereafter harvested by scraping the cells (Fig. 8). On close examination, such gold particles were associated with a 10–30-nm surface layer of homogenous structure, and with a patchy distribution, on the outer aspect of the cell membrane. Cellular projections and microvilli displayed a richer labeling than smooth parts of the cell surface. In addition, labeling was obvious along mitochondrial cristae, in association with Golgi complexes, particularly Golgi stack neighboring vesicles, and some endoplasmic reticulum strands, and vesicles just beneath the cell membrane. Observations on enzymatically released cells corroborated that the immunopositivity for the anti-sialyl-lactotetra antibody was uniform along the cell circumference (Fig. 9*A*). No cell surface labeling was observed when the sections were incubated with the anti-sialyl-neolactotetra antibody (Fig. 9*B*). Finally, the sulfatide binding antibody O4 gave no cell surface labeling (Fig. 9*C*). Instead, an intracellular labeling with distinct staining of mitochondria, along with endoplasmic reticulum and Golgi, was observed.

##### The Sialyl-lactotetra Epitope Is Also Present on hESC Glycoproteins

**FIGURE 10.**
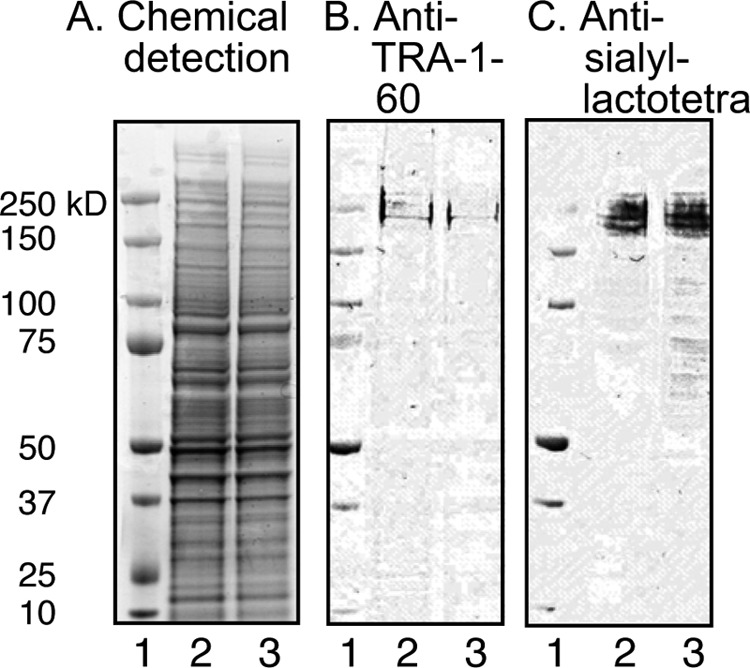
**Binding of anti-sialyl-lactotetra antibodies to protein extracts of hESC.** hESC protein extracts were separated on 4–12% Bis-Tris gels and subjected to staining with Imperial^TM^ protein stain (*A*), binding of anti-TRA-1–160 monoclonal antibodies (positive control) (*B*), and binding of anti-sialyl-lactotetra monoclonal antibodies (*C*). *Lane 1*, molecular mass standards; *lane 2*, protein extract of hESC line SA121, 13 μg; *lane 3*, protein extract of hESC line SA181, 13 μg.

Having established that the sialyl-lacotetra carbohydrate sequence is present on the cell surface of hESC, the binding of the anti-sialyl-lactotetra antibody to protein extracts of the hESC lines SA121 and SA181 was next examined. Thereby, a binding to a four distinct protein bands migrating at a molecular mass of ∼250 kDa was obtained (Fig. 10*C*, *lanes 2* and *3*). Thus, the sialyl-lactotetra epitope is present on both glycosphingolipids and glycoproteins of hESC lines.

##### Sialyl-lactotetra Is Down-regulated upon hiPSC Differentiation

**FIGURE 11.**
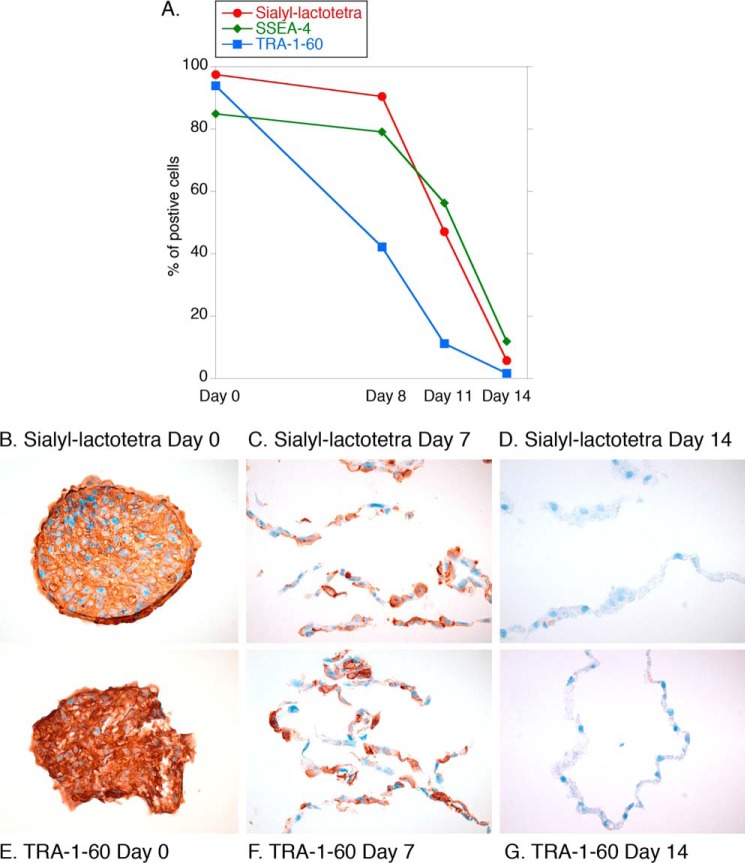
**Down-regulation of the sialyl-lactotetra epitope upon differentiation into hepatocyte-like cells.** *A*, flow cytometry analysis of hiPSC before and after induction of differentiation into hepatocyte-like cells. Expression of sialyl-lactotetra, SSEA-4, and TRA-1–60 was evaluated at days 0, 8, 11, and 14 after induction of differentiation the hiPSC line ChiPSC-4 into hepatocyte-like cells. Immunohistochemical investigation of hiPSC before and after induction of differentiation into hepatocyte-like cells. Expression of sialyl-lactotetra (*B–D*) and TRA-1–60 (*E–G*) was evaluated at days 0, 7, and 14 after induction of differentiation of the hiPSC line ChiPSC-4 into hepatocyte-like cells. Both stem cell markers are strongly positive on day 0 (*B* and *E*), and on day 7 the majority of cells are positive (*C* and *F*), whereas there is no staining on day 14 (*D* and *G*).

Next we followed sialyl-lactotetra expression during the course of differentiation of hiPSC into hepatocyte-like cells by flow cytometry and immunohistochemistry using the anti-sialyl-lactotetra antibody. During stem cell differentiation, the sialyl-lactotetra-positive cells drastically decreased from 97.5% at day 0 to 47.1% at day 11, and 5.79% at day 14 after induction of differentiation (Fig. 11*A*), as estimated by flow cytometry analysis. Similar differentiation-dependent reductions were observed for the established stem cell markers SSEA-4 and TRA-1–60.

Also, immunohistochemistry showed a gradual loss of sialyl-lactotetra upon differentiation. The reduction in staining intensity was first found on the fourth day (not shown), and thereafter the staining rapidly decreased, with loss staining in ∼20% of cells after 7 days (Fig. 11*C*), and after differentiation for 14 days, no intra- or extracellular staining was observed (Fig. 11*D*). The loss of sialyl-lactotetra upon differentiation was in parallel with the reduced expression of the established pluripotency marker TRA-1–60 (Fig. 11, *E–G*).

## DISCUSSION

There are great expectations placed on human pluripotent stem cells as a renewable source of cells for use in regenerative medicine. However, one important hurdle to the therapeutic application of differentiated human stem cells is the risk of teratoma formation by contaminating undifferentiated hPSC. Thus, for therapeutic development there is a need for tools to distinguish pluripotent from differentiated cells. Here, we identify the sialyl-lactotetra carbohydrate epitope as a novel marker of undifferentiated human pluripotent stem cells. Sialyl-lactotetraosylceramide was initially characterized as an acid glycosphingolipid of hESC lines, not present hESC-derived hepatocyte-like and cardiomyocyte-like cells. A high expression of sialyl-lactotetra on the cell surface of hPSC that concurrently expressed established stem cell markers was shown by flow cytometry and immunohistochemistry and confirmed by electron microscopy. Thereby, sialyl-lactotetra was also detected in endoplasmic reticulum and Golgi, in agreement with the route for glycosphingolipid biosynthesis, and the presence of sialyl-lactotetra in mitochondria was also established.

After induction of differentiation of hiPSC lines into hepatocyte-like cells, the established pluripotency markers TRA-1–60 and SSEA-4 followed the kinetics of disappearance reported by Ramirez *et al.* (22), and the expression of sialyl-lactotetra also rapidly diminished and had almost completely disappeared after 14 days. These findings further support the notion that sialyl-lactotetra down-regulation accompanies the loss of pluripotency and indicates an early differentiation state of stem cells.

In normal human tissues, the distribution of sialyl-lactotetraosylceramide is very limited. Sialyl-lactotetraosylceramide has only been found in human meconium (23) and in the brains of young children, where it gradually disappears after two years age (17). In addition, sialyl-lactotetraosylceramide has been found in some human cancers, such as small cell lung carcinoma (24) and glioma (25, 26), and in embryonal carcinoma cells (27).

An investigation of the total cellular glycome of hESC and hiPSC was recently reported (9). One of the gangliosides characterized therein was sialyl-neolactotetraosylceramide, which was not found in our case. One possible explanation for this discrepancy is that the sialyl-neolactotetraosylceramide observed by Fujitani *et al.* (9) was derived during culture of the hESC and hiPSC. Culture of hESC in the presence of animal-derived compounds leads to uptake of compounds, such as the nonhuman sialic acid NeuGc (28, 29). Uptake of NeuGc may also explain the high expression of the nonhuman ganglioside NeuGc-GM1 in hESC reported by Fujitani *et al.* (9). However, the hESC lines used here for isolating glycosphingolipids were grown in a serum- and cell-free system, and thus there are no contaminating compounds of animal origin.

Another ganglioside of potential interest as hPSC marker is sialyl-globotetraosylceramide. This ganglioside has only been characterized in human teratocarcinoma cells (30) and in muscles affected by amyothropic lateral sclerosis (31). However, further studies of sialyl-globotetraosylceramide as a potential hPSC marker must await the development of specific reagents for this compound.

Sulfated glycosphingolipids have not previously been described in hPSC. However, in the previous studies of hESC glycosphingolipids, the mass spectrometric analyses were done using permethylated derivatives (7, 8). Sulfated glycosphingolipids were not found in these studies, because sulfate groups are lost upon permethylation.

Sulfatide, and to some extent sulf-lactosylceramide, are present in a variety of human tissues, such as the brain and the gastrointestinal tract (reviewed in Ref. 32). Sulfatide is also present in a number of human cancers, and abnormal sulfatide metabolism has been associated with several diseases, such as autoimmune diseases including diabetes mellitus (32).

Although sulfatide and sulf-lactosylceramide were present in the acid glycosphingolipid fractions of the hESC lines, these compounds were not detected on the cell surface when using the anti-SO_3_-3Galβ antibody in flow cytometry, and using immunohistochemistry only a faint cytoplasmic staining was obtained. Interestingly, the electron microscopy, having a higher sensitivity, demonstrated a distinct staining of mitochondria, along with endoplasmic reticulum and Golgi, by the anti-sulfatide antibodies. This sulfatide binding antibody O4 is a marker of immature oligodendrocytes (33) and has been used to follow the differentiation of hPSC into oligodendrocytes (34–36). However, our findings demonstrate that sulfatide is produced already in undifferentiated hESC lines but is retained within intracellular compartments. The mechanism for mobilization of sulfatide to the cell membrane upon oligodendrocyte differentiation should be further investigated.

An additional observation is the distinct binding of the anti-sialyl-lactotetra antibody to the two reference acid glycosphingolipid fractions of human cancers in Fig. 1*C* (human hepatoma and liver metastasis of human lung cancer). According to the cancer stem cell theory, cancers are maintained by subpopulations of tumor cells with stem or progenitor cell characteristics expressing stem cell-associated markers, and it is these cells that initiate tumor formation and differentiate along multipotent pathways (37). An important next step is thus to investigate whether it is possible to identify and isolate cancer-initiating cells using the sialyl-lactotetra marker.

In summary, herein we show that the sialyl-lactotetra carbohydrate sequence is specifically expressed in undifferentiated pluripotent stem cell lines, both hESC and hiPSC, and its expression decreases upon early differentiation coincidently with a down-regulation of the established stem cell markers SSEA-4 and TRA-1–60. These features make sialyl-lactotetra a much needed addition to the collection of markers of hPSC. Such marker has a huge potential for use in quality control assays for cell therapy programs, can be used for negative selection of undifferentiated stem cells in therapeutic cell products, and may shed new light on the relation between cancer stem cells and hPSC.

## Supplementary Material

Supplemental Data

## Footnotes

6 The glycosphingolipid nomenclature follows the recommendations by the IUPAC-IUB Commission on Biochemical Nomenclature (CBN for Lipids, *Eur. J. Biochem*. (1998) **257,** 293). It is assumed that Gal, Glc, GlcNAc, GalNAc, NeuAc and NeuGc are of the D-configuration, Fuc is of the L-configuration, and all sugars are present in the pyranose form.

7 In the shorthand nomenclature for fatty acids and bases, the number before the colon refers to the carbon chain length, and the number after the colon gives the total number of double bonds in the molecule. Fatty acids with a 2-hydroxy group are denoted by the prefix h before the abbreviation, *e.g.* h16:0. For long chain bases, d denotes dihydroxy, and t denotes trihydroxy. Thus, d18:1 indicates sphingosine (1,3-dihydroxy-2-aminooctadecene), and t18:0 indicates phytosphingosine (1,3,4-trihydroxy-2-aminooctadecane).

## Footnotes

5 The abbreviations used are:

hESC: human embryonic stem cells
ESI: electrospray ionization
hiPSC: human induced pluripotent stem cells
hPSC: human pluripotent stem cells
SSEA: stage-specific embryonic antigen.
