# Supplementary material for: Sialyl-lactotetra, a Novel Cell Surface Marker of Undifferentiated Human Pluripotent Stem Cells
Source: J Biol Chem. 2014 May 19;289(27):18846–59. doi: 10.1074/jbc.M114.568832 (PMC4081926; doi:10.1074/jbc.M114.568832)
Supplement: Supplemental Data [file supp_289_27_18846__index.html]

Sialyl-lactotetra, a Novel Cell Surface Marker of Undifferentiated Human Pluripotent Stem Cells — Sialyl-lactotetra, a Novel Stem Cell Marker — Supplemental Data 

# Sialyl-lactotetra, a Novel Cell Surface Marker of Undifferentiated Human Pluripotent Stem Cells

## Supplemental Data

**Files in this Data Supplement:**

- Supplemental Table S1 (.pdf, 79 KB) - Supplemental Table S1
- Supplemental Table S2
