# Supplementary material for: Sialyl-lactotetra, a Novel Cell Surface Marker of Undifferentiated Human Pluripotent Stem Cells
Source: J Biol Chem. 2014 May 19;289(27):18846–59. doi: 10.1074/jbc.M114.568832 (PMC4081926; doi:10.1074/jbc.M114.568832)
Supplement: Supplemental Data [file supp_M114.568832_jbc.M114.568832-1.pdf]

Table S1. Glycosphingolipid structures mentioned in this study

Trivial name	Structure
I. Non-acid glycosphingolipids	
GalCer	Galβ1Cer
GlcCer	Glcβ1Cer
LacCer	Galβ4Glcβ1Cer
Galabia	Galα4Galβ1Cer
Lactotri	GlcNAcβ3Galβ4Glcβ1Cer
Gangliotri/asialoGM2	GalNAcβ4Galβ4Glcβ1Cer
Globotri	Galα4Galβ4Glcβ1Cer
Globotetra	GalNAcβ3Galα4Galβ4Glcβ1Cer
Globopenta/SSEA-3	Galβ3GalNAcβ3Galα4Galβ4Glcβ1Cer
Globo H	Fucα2Galβ3GalNAcβ3Galα4Galβ4Glcβ1Cer
Lactotetra	Galβ3GlcNAcβ3Galβ4Glcβ1Cer
Fucosyl-lactotetra/H type 1 penta	Fucα2Galβ3GlcNAcβ3Galβ4Glcβ1Cer
H type 2 penta	Fucα2Galβ4GlcNAcβ3Galβ4Glcβ1Cer
Le^{x} penta	Galβ4(Fucα3)GlcNAcβ3Galβ4Glcβ1Cer
Le^{y} hexa	Fucα2Galβ4(Fucα3)GlcNAcβ3Galβ4Glcβ1Cer
A type 1 hexa	GalNAcα3(Fucα2)Galβ3GlcNAcβ3Galβ4Glcβ1Cer
II. Acid glycosphingolipids	
GM3	NeuAcα3Galβ4Glcβ1Cer
GM2	GalNAcβ4(NeuAcα3)Galβ4Glcβ1Cer
GM1	Galβ3GalNAcβ4(NeuAcα3)Galβ4Glcβ1Cer
NeuGcGM1	Galβ3GalNAcβ4(NeuGcα3)Galβ4Glcβ1Cer
GD3	NeuAcα8NeuAcα3Galβ4Glcβ1Cer
GD1a	NeuAcα3Galβ3GalNAcβ4(NeuAcα3)Galβ4Glcβ1Cer
GD1b	Galβ3GalNAcβ4(NeuAcα8NeuAcα3)Galβ4Glcβ1Cer
Sialyl-Le^{a}	NeuAcα3Galβ3(Fucα4)GlcNAcβ3Galβ4Glcβ1Cer

Sialyl-Le^x	NeuAcα3Galβ4(Fucα3)GlcNAcβ3Galβ4Glcβ1Cer
Sialyl-globopenta/SSEA-4	NeuAcα3Galβ3GalNAcβ3Galα4Galβ4Glcβ1Cer
Disialyl-globopenta	NeuAcα3Galβ3(NeuAcα6)GalNAcβ3Galα4Galβ4Glcβ1Cer
Sialyl-lactotetra	NeuAcα3Galβ3GlcNAcβ3Galβ4Glcβ1Cer
Sialyl-neolactotetra	NeuAcα3Galβ4GlcNAcβ3Galβ4Glcβ1Cer
Sialyl-globotetra	NeuAcα3GalNAcβ3Galα4Galβ4Glcβ1Cer
III. Sulfated glycosphingolipids	
Sulfatide	SO_3-3Galβ1Cer
Sulf-LacCer	SO_3-3Galβ4Glcβ1Cer
Sulf-globopenta	SO_3-3Galβ3GalNAcβ3Galα4Galβ4Glcβ1Cer
HNK-1	SO_3-3GlcAβ3Galβ4GlcNAcβ3Galβ4Glcβ1Cer
