# Supplementary material for: Sialyl-lactotetra, a Novel Cell Surface Marker of Undifferentiated Human Pluripotent Stem Cells
Source: J Biol Chem. 2014 May 19;289(27):18846–59. doi: 10.1074/jbc.M114.568832 (PMC4081926; doi:10.1074/jbc.M114.568832)
Supplement: Supplemental Data [file supp_M114.568832_New_Table_S2.pdf]

Table S2. Monoclonal antibodies used in chromatogram binding assays.

Antibody	Clone/Designation	Manufacturer/Reference	Dilution	Isotype	hESC binding[1]
Anti-SSEA-4	MC-813-70	eBioScience	1:100	IgG3	+
Anti-sialyl-Lewis^x	KM93	Merck	1:50	IgM	-
Anti-sialyl-Lewis^a	116-NS-19-9	Signet	1:30	IgG1	-
Anti-GD1a	GD1a-1	Millipore	1:100	IgG1	-
Anti-HNK-1	VC1.1	Sigma	1:50	IgM	-
Anti-SO_3-3Galβ	Sulph-1	(1)[2]	1:100	IgG1	+
Anti-GM1	GM1:1	(2)	1:50	IgM	-
Anti-GM2	DMAb-1	(3)	1:50	IgM	-
Anti-GD3	DMAb-7	(4)	1:100	IgM	-
Anti-sialyl-lactotetra	TR4/SL-50	(5)	1:1000	IgM	+
Anti-di-sialyl-lactotetra	DMAb22	(6)	1:2000	IgM	-
Anti-NeuAcα3-neolactotetra	LM1:1a	(7)	1:1000	IgM	-
Anti-NeuAcα6-neolactotetra	LM4:2	(8)	1:100	IgG	-

[1]Binding is defined as follows: + denotes a binding when 40 μg of the acid glycosphingolipid fraction of hESC SA181 or SA121 was applied on the thin-layer chromatogram, while - denotes no binding.
[2]References:

1. Fredman, P., Mattson, L., Andersson, K., Davidsson, P., Ishizuka, I., Jeansson, S., Månsson, J.-E., and Svennerholm, L. (1988) Characterization of the binding epitope of a monoclonal antibody to sulphatide. *Biochem. J.* **251**, 17-22
2. Molander, M., Berthold, C.H., Persson, H., Andersson, K., and Fredman, P. (1997) Monosialoganglioside (GM1) immunofluorescence in rat spinal roots studied with a monoclonal antibody. *J. Neurocytol*. **26**, 101-111
3. Karlsson, G., Månsson, J.E., Wikstrand, C., Bigner, D., and Svennerholm, L. (1990) Characterization of the binding epitope of the monoclonal antibody DMAb-1 to ganglioside GM2. *Biochim. Biophys. Acta* **1043**, 267-272
4. He, X., Wikstrand, C.J., Fredman, P., Månsson, J.E., Svennerholm, L., and Bigner, D.D. (1989) GD3 expression by cultured human tumor cells of neuroectodermal origin. *Acta. Neuropathol*. **79**, 317-325
5. Wikstrand, C.J., Longee, D.C., McLendon, R.E., Fuller, G.N., Friedman, H.S., Fredman, P., Svennerholm, L., and Bigner, D.D. (1993) Lactotetraose series ganglioside 3'6'-isoLD1 in tumors of central nervous and other systems *in vitro* and *in vivo*. *Cancer Res*. **53,** 120-126
6. Svennerholm, L., Boström, K., Fredman, P., Månsson, J.-E., Rosengren, B., and Rynmark, B.-M. (1989) Human brain gangliosides: developmental changes from early fetal stage to advanced age. *Biochim. Biophys. Acta* **1005**, 109-117
7. Svennerholm, L., Rynmark, B.M., Vilbersson, G., Fredman, P., Gottfries, J., Månsson, J.-E., and Percy, A. (1991) Gangliosides in human fetal brain. *J. Neurochem*. **56**, 1763-1768
8. Nilsson, O., Lindholm, L., Holmgren, J. and Svennerholm, L. (1985) Monoclonal antibodies raised against NeuAcα2-6neolactotetraosylceramide detect carcinoma-associated gangliosides. *Biochim. Biophys. Acta* **835**, 577-583
